# Priority order of neonatal colonization by a probiotic or pathogenic *Escherichia coli* strain dictates the host response to experimental colitis

**DOI:** 10.3389/fmicb.2024.1393732

**Published:** 2024-08-14

**Authors:** Tomas Hudcovic, Petra Petr Hermanova, Hana Kozakova, Oldrich Benada, Olga Kofronova, Martin Schwarzer, Dagmar Srutkova

**Affiliations:** ^1^Laboratory of Gnotobiology, Institute of Microbiology of the Czech Academy of Sciences, Nový Hrádek, Czechia; ^2^Laboratory of Molecular Structure Characterization, Institute of Microbiology of the Czech Academy of Sciences, Prague, Czechia

**Keywords:** *Escherichia coli*, priority effect, DSS-experimental colitis, mouse model, immune modulation

## Abstract

The alarming prevalence of inflammatory bowel disease (IBD) in early childhood is associated with imbalances in the microbiome, the immune response, and environmental factors. Some pathogenic *Escherichia coli* (*E. coli)* strains have been found in IBD patients, where they may influence disease progression. Therefore, the discovery of new harmful bacterial strains that have the potential to drive the inflammatory response is of great importance. In this study, we compared the immunomodulatory properties of two *E. coli* strains of serotype O6: the probiotic *E. coli* Nissle 1917 and the uropathogenic *E. coli* O6:K13:H1. Using the epithelial Caco-2 cell line, we investigated the different abilities of the strains to adhere to and invade epithelial cells. We confirmed the potential of *E. coli* Nissle 1917 to modulate the Th1 immune response in a specific manner in an *in vitro* setting by stimulating mouse bone marrow-derived dendritic cells (BM-DCs). In gnotobiotic *in vivo* experiments, we demonstrated that neonatal colonization with *E. coli* Nissle 1917 achieves a stable high concentration in the intestine and protects mice from the progressive effect of *E. coli* O6:K13:H1 in developing ulcerative colitis in an experimental model. In contrast, a single-dose treatment with *E. coli* Nissle 1917 is ineffective in achieving such high concentrations and does not protect against DSS-induced ulcerative colitis in mice neonatally colonized with pathobiont *E. coli* O6:K13:H1. Despite the stable coexistence of both *E. coli* strains in the intestinal environment of the mice, we demonstrated a beneficial competitive interaction between the early colonizing *E. coli* Nissle 1917 and the late-arriving strain O6:K13:H1, suggesting its anti-inflammatory potential for the host. This study highlights the importance of the sequence of bacterial colonization, which influences the development of the immune response in the host gut and potentially impacts future quality of life.

## Introduction

The mucosal surfaces of newborns, including the gastrointestinal tract, undergo dynamic adaptation to the microbial world during the first stages of life, with bacteria playing a dominant role. The first colonizing microbiota educates the immune system, and the nature and order of these bacteria are considered crucial for future health, with long-term consequences for the host (Gensollen et al., 2016). Early-arriving microbes exhibit enhanced colonization stability and have the most significant influence on the microbial community, suggesting that the timing of bacterial arrival in the gut is key to shaping the gut microbiome (Martínez et al., 2018).

The outcome of competitive interactions between arriving microorganisms (coexistence or exclusion of competition) also determines the order in which species arrive in an ecosystem, leading to a phenomenon known as the “priority effect” (Fukami, 2015; Sprockett et al., 2018). Priority effects occur in the gut when: (i) the first colonizing species either occupy or modify the ecological niche (e.g., consume certain nutrients), inhibiting or facilitating later arriving species; (ii) late arriving species have high environmental requirements; or (iii) early and late-arriving species have a strong niche overlap (e.g., closely related taxa and/or taxa competing for the same resources).

Priority effects have been described in the gut ecosystem of gnotobiotic mouse models with isogenic bacterial strains of *Bacteroides* or *Akkermansia* species (Lee et al., 2013; Segura Munoz et al., 2022) or mouse fecal communities (Martínez et al., 2018). These ecological principles have also been demonstrated in the establishment of the intestinal microbiome in hospital-associated preterm infants (Rao et al., 2021). However, the specific impact of isogenic bacterial strains of the same species on the host immune response in intestinal inflammation has not yet been investigated.

Inflammatory bowel diseases (IBD) encompass a group of chronic, non-specific, recurrent, immune-mediated inflammations of the gastrointestinal tract. These are divided into similar yet distinct subtypes: Crohn's disease (CD), pouchitis, and ulcerative colitis (UC) (Sartor and Muehlbauer, 2007). The incidence of these diseases has increased dramatically in Western and industrialized countries, suggesting a link between modern lifestyles and environmental factors. Notably, the high prevalence of IBD in early childhood has been recently documented (Park et al., 2020).

These diseases have a multifactorial etiology, combining genetic predisposition and environmental factors such as dysbiotic microbiome, poor dietary habits, stress, impaired intestinal epithelial integrity, dysregulated immune response against commensal gut microbes, and other factors (Zhang and Li, 2014). Although the exact molecular mechanisms of pathogenesis are not yet clear, there is a strong consensus that the intestinal microbiota plays a central role in the development of IBD (Nishida et al., 2018). Current research focuses on the dynamic balance between commensal gut bacteria and host defense mechanisms at the intestinal mucosa in the initiation and maintenance of intestinal inflammation. To date, no specific microbe has been shown to cause IBD, although several specific microorganisms have been investigated as potential factors in IBD etiopathogenesis (Damaskos and Kolios, 2008).

Interestingly, recent research has shown both a decreased diversity of the microbiota and an increased frequency of virulence markers primarily associated with *E. coli* in IBD (Martinez-Medina and Garcia-Gil, 2014; Moustafa et al., 2018). The majority of IBD patients have been shown to exhibit increased positive antibody responses to a variety of *E. coli* O antigens, including serotypes O1, O2, O6, O18, and O75. These strains have mostly been associated with urinary tract infections and/or originate from the fecal microbiota (Tabaqchali et al., 1978; Petersen et al., 2009; Mirsepasi-Lauridsen et al., 2019). Epithelial-associated invasive *E. coli* strains have been frequently isolated from the ileal and colonic mucosa of CD patients, where they can proliferate and damage the host tissue (Boudeau et al., 1999).

Mirsepasi-Lauridsen et al. (2016) showed that UC-associated *E. coli* p19A, an extraintestinal pathogenic *E. coli* (ExPEC) strain containing α-hemolysin, disrupts the tight junction (TJ) protein occludin in Caco-2 cells *in vitro*, resulting in increased barrier permeability. In addition, the UC-associated strain p19A induces cell death in dendritic cells and stimulates the release of the proinflammatory cytokines TNF-α, IL-6, and IL-23 (Jensen et al., 2015).

New and emerging therapies for IBD are currently being researched and developed to target immunoregulatory processes. Probiotics and their metabolites are crucial for gut homeostasis, suggesting that they may play a role in the treatment of IBD (Yoshimatsu et al., 2021).

*E. coli* Nissle 1917 (EcN; O6:K5:H1) is probably the best-studied probiotic strain. It was originally isolated by Alfred Nissle during the First World War in search of wild *E. coli* strains with antagonistic activity against enteric pathogens (Sonnenborn, 2016). Based on genome analysis, it is currently known that this probiotic strain lacks several defined virulence factors (e.g., α-hemolysin, P-fimbrial adhesins, and the semi-rough lipopolysaccharide phenotype) and expresses fitness factors such as microcins, various iron uptake systems, adhesins, and proteases (Grozdanov et al., 2004). Schlee et al. (2007) showed that *E. coli* Nissle 1917 induces the expression of human β-defensin 2, an antimicrobial peptide that contributes to the strengthening of the intestinal mucosal barrier by limiting bacterial adherence and bacterial invasion of the gut mucosa.

The protective effect of EcN against infections with pathogenic *Salmonella enterica, Yersinia enterocolitica, Shigella flexneri, Listeria monocytogenes, Vibrio cholerae*, and host cell invasion by adherent-invasive *E. coli* (AIEC) has been described using *in vitro* and *in vivo* models (Boudeau et al., 2003; Altenhoefer et al., 2004; Splichalova et al., 2011; Nag et al., 2018; Splichal et al., 2019).

EcN also strengthens the intestinal epithelial barrier by supporting tight junctions (TJs) between intestinal epithelial cells and modulating the host immune response toward an anti-inflammatory balance (Zyrek et al., 2007; Trebichavsky et al., 2010). Several clinical studies have highlighted its remarkable therapeutic effects, especially in maintenance of remission in UC patients (Schultz, 2008). Similarly, the anti-inflammatory effect of this probiotic on the gut has been demonstrated in some experimental colitis models or during the recovery phase from induced colitis in mice and rats (Grabig et al., 2006; Souza et al., 2016; Rodríguez-Nogales et al., 2018; Lu et al., 2019; Park et al., 2021).

In our previous study, we showed that BALB/c mice monocolonized with the probiotic strain *E. coli* Nissle 1917 did not develop dextran sulfate sodium (DSS)-induced colitis compared to mice monocolonized with the uropathogenic strain *E. coli* O6:K13:H1, which developed similar inflammatory changes as conventionally reared mice (Hudcovic et al., 2007). Interestingly, we found that the two strains, *E. coli* Nissle 1917 (O6:K5:H1) and *E. coli* O6:K13:H1, induce an almost identical gene signature in the small intestine or organoids of monocolonized mice (Janeckova et al., 2019).

In the present study, we focus on the comparative analysis of the immunomodulatory properties of these two strains using *in vitro* and *in vivo* experiments. Using the epithelial Caco-2 cell line, we investigated the different abilities of the strains to adhere to and invade epithelial cells. Additionally, using dendritic cells isolated from the bone marrow of mice, we confirmed the potential of *E. coli* Nissle 1917 to modulate Th1 immune responses.

In gnotobiotic *in vivo* experiments, we demonstrated that neonatal colonization with *E. coli* Nissle 1917 induces colonization resistance against *E. coli* O6:K13:H1 and protects mice against the development of experimentally induced intestinal inflammation. Conversely, in mice neonatally colonized with the pathobiont *E. coli* O6:K13:H1, a single administration of *E. coli* Nissle 1917 was inefficient in achieving a high concentration in the intestine, as observed with neonatal colonization, and it did not protect the mice from developing acute ulcerative colitis, demonstrating the absolute importance of the order in which intestinal colonizing strains are introduced.

## Materials and methods

### Bacterial strains

The *E. coli* Nissle 1917 (EcN; O6:K5:H1) strain was purchased as the probiotic drug Mutaflor (Ardeypharm, Herdecke, Germany). The *E. coli* O6:K13:H1 (EcO) was originally isolated from the urine of a patient with cystitis and was kindly provided by Prof. L. A. Hanson (Department of Immunology, Institute of Medical Microbiology, University of Gothenburg, Sweden). For *in vitro* adhesivity and invasivity analysis, the adherent-invasive *E. coli* LF82 strain (a gift from Arlette Darfeuille-Michaud, France) was used as the positive control (Boudeau et al., 2003).

### Bacteria preparation

All *E. coli* strains were cultivated in Luria-Bertani (LB) broth (Himedia Laboratories, USA) overnight at 37°C under aerobic conditions. For *in vitro* adhesivity and invasivity assays on the Caco-2 cell line, a concentration of 2 x 10^7^ CFU/ml in PBS was used.

Formalin-inactivated bacteria were used for *in vitro* stimulation of human embryonic kidney cells and bone marrow-derived dendritic cells (BM-DCs), as previously described (Srutkova et al., 2015). Briefly, bacterial cells were plated on LB agar (Himedia Laboratories, USA) to determine the concentration of the bacterial suspension before inactivation. Bacteria were then centrifuged, washed with phosphate-buffer saline (PBS), and inactivated with 1% formalin in phosphate-buffer for 3 h at room temperature. After inactivation, cells were centrifuged, washed three times with PBS, adjusted to 1 x 10^9^ CFU/ml in PBS, and stored at 4°C.

For the *in vivo* study, EcN or EcO strains were cultivated overnight in LB broth at 37°C under aerobic conditions, centrifuged (4000 RPM for 10 min at 4°C), and diluted in PBS according to optical density to an approximate concentration of 0.5–1.0 x 10^9^ CFU/ml. The final *E. coli* concentration administered to the mice was determined by bacteria plating on LB agar as 1.9 x 10^8^ CFU/0.2 ml for EcN and 1.1 x 10^8^ CFU/0.2 ml for EcO, respectively.

### Scanning electron microscopy (SEM)

EcN and EcO strains grown in 5 ml of LB broth at 37°C overnight were processed as follows: The bacteria were fixed in 3% glutaraldehyde with cacodylate buffer for 15 min at room temperature, followed by overnight incubation at 4°C. The extensively washed cells were sedimented overnight onto poly-L-lysine-treated circular coverslips at 4°C. The coverslips with the bacteria were then post-fixed in 1% OsO_4_ for 1 h at room temperature.

The washed coverslips were dehydrated through a graded alcohol series (25%, 50%, 75%, 90%, 96%, 100%, and 100%; each step for 20 min), followed by absolute acetone, and then dried from liquid CO_2_ in a K850 Critical Point Dryer (Quorum Technologies Ltd., Ringmer, UK). The dried samples were sputter-coated with a 3-nm layer of platinum in a Q150T Turbo-Pumped Sputter Coater (Quorum Technologies Ltd., Ringmer, UK). The final specimens were examined using CBS and TLD detectors in an FEI Nova NanoSEM scanning electron microscope (FEI, Brno, Czechia) at 5 kV (Benada and Pokorny, 1990; Klimentova et al., 2019).

### Transmission electron microscopy (TEM)

The EcN and EcO strains were grown in 5 ml of LB broth at 37°C until the exponential phase for 3 h. Then, the bacteria were negatively stained on glow-discharge-activated formvar/carbon grids (Benada and Pokorny, 1990) with 1% ammonium molybdate supplemented with 0.1% trehalose (Harris and Scheffler, 2002). The final samples were viewed in a Philips CM100 electron microscope (FEI, formerly Philips EO, The Netherlands) at 80 kV. Digital images were recorded using a Veleta slow-scan camera (EMSis, Muenster, Germany) at a magnification of 25,000 × or 46,000 × and processed with AnalySis 5.2 software (Olympus Soft Imaging Solutions GmbH, Muenster, Germany).

### Caco-2 cell line culture

The human epithelial colorectal adenocarcinoma cell line (Caco-2) was kindly provided by Zuzana Jiraskova Zakostelska (Institute of Microbiology of the Czech Academy of Sciences). Cells were cultured in Dulbecco's Modified Eagles high glucose, supplemented with 10% fetal calf serum (FCS), 0.01 M HEPES, 1% penicillin/streptomycin, 1% non-essential amino acids, and 2 mM L-glutamine (all Sigma-Aldrich, CO., St. Louis, USA). The cells were routinely cultured in a 75 cm^2^ tissue culture flask (Techno Plastic, Trasadingen, Switzerland) at 37°C in a humidified atmosphere with 5% CO_2_. The medium was changed every second or third day, and cells were re-seeded with trypsin (Sigma-Aldrich, CO., St. Louis, USA) when the monolayer reached 80% confluence. The cell line between the 25th and 30th passages was used for analysis.

### Adhesive and invasive properties of *E. coli* strains determined by transwell culture of the Caco-2 cell line

Caco-2 cells at a concentration of 2 x 10^4^ per 100 μl of media were added to the apical chamber, and 500 μl of complete media was added to the basolateral chamber of the transwell insert in a 24-well plate (Corning, Sigma-Aldrich, CO., St. Louis, USA). The cells were cultured for 21 days, with medium changes in both chambers every second or third day. Afterward, the medium was removed, and the cells were washed two times with PBS to remove any remaining antibiotics.

Live bacteria at a concentration of 2 x 10^6^ CFU in 100 μl of antibiotic-free medium were added to the apical part, and 500 μl of antibiotic-free medium was added to the basolateral chamber. After 5 h of incubation at 37°C in a humidified atmosphere with 5% CO_2_, both chambers were washed three times with 2 ml of PBS.

To determine the adhesive properties of the bacteria, 100 μl of 0.1% Triton-X100 (Sigma-Aldrich) was added to the apical chamber, and the cells with adhered bacteria were harvested by pipetting up and down. The cells were then plated onto Petri dishes with LB agar to determine the number of bacteria adhered to the cells.

To detect invasive properties, transwell inserts with invaded bacteria were washed three times with PBS and then incubated with culture media supplemented with 2% penicillin, streptomycin, and gentamycin (all Sigma-Aldrich) to eliminate adhered bacteria. After 1 hour of incubation at 37°C, the transwell inserts were washed three times using PBS, and the cells were harvested using 100 μl of 0.1% Triton-X100. The cells were plated on LB agar. The results from three independent experiments are pooled and expressed as a percentage of adhered or invaded bacteria from the input bacterial concentrations.

### Stimulation of human embryonic kidney 293 cells stably transfected with TLR4, TLR2/CD14-, and NOD2

Human embryonic kidney (HEK) 293 cells stably transfected with plasmid carrying human gene TLR4/MD2/CD14 (a gift from Prof. M. Yazdanbakhsh, Leiden, Netherlands), TLR2/CD14- (InvivoGen, USA), or hNOD2 (InvivoGen, USA) were used to test pattern recognition receptor modulatory analysis (Schwarzer et al., 2013). Briefly, cells (2 × 10^5^ cells/ml) were cultured in Dulbecco's complete medium (Sigma-Aldrich) supplemented with appropriate antibiotics (Blasticidin and Hygromycin B Gold from Invivogen, USA) in a final volume of 200 ul per well in a 96-well plate, according to the manufacturer's recommendation. Once the cells adhered to the bottom of the well, EcN or EcO at concentrations of 1 x 10^8^, 1 x 10^7^, and 1 x 10^6^ CFU/ml were added.

hTLRs and hNOD2 agonists were used as a positive control to activate selected signaling pathways: lipopolysaccharide (LPS-EB, 1 μg/ml, InvivoGen, USA) for the HEK 293/hTLR4 pathway, synthetic triacylated lipopeptide PAM3CSK4 (PAM3, 1 μg/ml, InvivoGen, USA) for the HEK 293/hTLR2CD14- pathway, and muramyl dipeptide (MDP, 1 μg/ml, InvivoGen, USA) for the HEK 293/hNOD2 pathway. Cultivation medium was used as a negative control.

Culture supernatants were harvested after 20 h of stimulation with formalin-inactivated EcN and EcO, and the cytokine level of human interleukin 8 (IL-8) was measured by ELISA according to the manufacturer's recommendation (Invitrogen, Thermo Fisher Scientific, USA). Each experiment was repeated at least four times.

### Immunomodulatory properties of EcN and EcO in bone marrow-derived dendritic cells (BM-DCs)

BM-DC were isolated from naïve BALB/c mice (8 weeks old, *n* = 2) and prepared as previously described (Srutkova et al., 2015). Briefly, bone marrow precursors isolated from femurs and tibias were seeded at 2 × 10^7^ cells/ml in RPMI 1640 culture medium (Sigma-Aldrich) containing 10% FBS, penicillin/streptomycin, 0.01 M HEPES, and 20 ng/ml mouse GM-CSF (eBioscience, USA) and incubated for 8 days at 37°C in an atmosphere with 5% CO_2_. Fresh medium was added on days 3 and 6. Cells were then harvested, diluted to the final concentration of 2 × 10^6^ cells/ml, and stimulated with the formalin-inactivated EcN and EcO strains individually or in a mixture (1:1) at a concentration of 2 x 10^7^ CFU/ml for 18 h.

For stimulation using subsequently both bacteria, BM-DCs were cultivated with the first bacterial strain (2 x 10^7^ CFU/ml) for 5 h at 37°C in 5% CO_2_, washed with culture medium, and then the second bacterial strain was added and cultured for 13 h. Ultrapure LPS (1 μg/ml; InvivoGen, USA) was used as a positive control; unstimulated cells (medium) served as a negative control. The levels of IL-10 and IL-12p70 were determined using Ready-Set-Go! Kit (eBioscience, USA) according to the manufacturer's instructions.

For cell surface marker analysis, BM-DCs were labeled for 30 min at 4°C with anti-mouse FITC-conjugated CD11c, APC-conjugated MHC II, and PE-conjugated CD40, CD80, or CD86 monoclonal antibodies (eBioscience, USA). The data were acquired on a BD FACS Calibur flow cytometer (BD Biosciences, USA) and analyzed with FlowJo software 7.6.2 (TreeStar, USA).

### Animals

Germ-free (GF), *E. coli* Nissle 1917, or *E. coli* O6:K13:H1 monocolonized and experimental groups of BALB/c mice were kept under sterile conditions in Trexler-type plastic isolators in a room with a 12 h light-dark cycle at 22°C. They were fed a 50 kG irradiated sterile pellet diet (Altromin, Lage, Germany) and provided with sterile drinking water *ad libitum*.

Sterility and colonization status were controlled every 2 weeks by confirming the absence of unwanted bacteria, molds, and yeasts through aerobic and anaerobic cultivation of mouse feces and swabs from the isolators in VL (Viande-Levure), Sabouraud-dextrose, and meat-peptone broth, followed by plating on blood, Sabouraud, and VL agar plates.

Specific pathogen-free (SPF) mice were kept in individually ventilated cages (IVC) (Tecniplast S.P.A., Italy), with a 12 h light-dark cycle at 22°C, fed a 25 kG irradiated sterile pellet diet (Altromin, Lage, Germany), and provided with drinking water *ad libitum*. SPF mice were regularly checked for the absence of potential pathogens according to an internationally established standard (FELASA). Animal experiments were approved by the committee for the protection and use of experimental animals at the Institute of Microbiology of the Czech Academy of Science (approval ID: 18/2019).

### Experimental design

Germ-free (GF) mice were colonized by intragastric gavage with either *E. coli* Nissle 1917 (Mutaflor) or *E. coli* O6:K13:H1, and mated after a 2-week colonization period. Their offspring were used for *in vivo* studies.

Subsequently, 2-month-old female offspring were divided into experimental groups according to bacterial colonization and DSS-colitis induction:

1. neonatally monocolonized mice by *E. coli* O6:K13:H1 without the second colonization (EcO group, *n* = 6).
2. neonatally monocolonized by *E. coli* Nissle 1917 with the second colonization by intragastric gavage of *E. coli* O6:K13:H1 (healthy EcN+EcO group, *n* = 5; and EcN+EcO group, *n* = 6).
3. neonatally monocolonized by *E. coli* O6:K13:H1 with the second colonization by intragastric gavage of *E. coli* Nissle 1917 (healthy EcO+EcN group, *n* = 3; and EcO+EcN group, *n* = 4).

**Figure 1:**
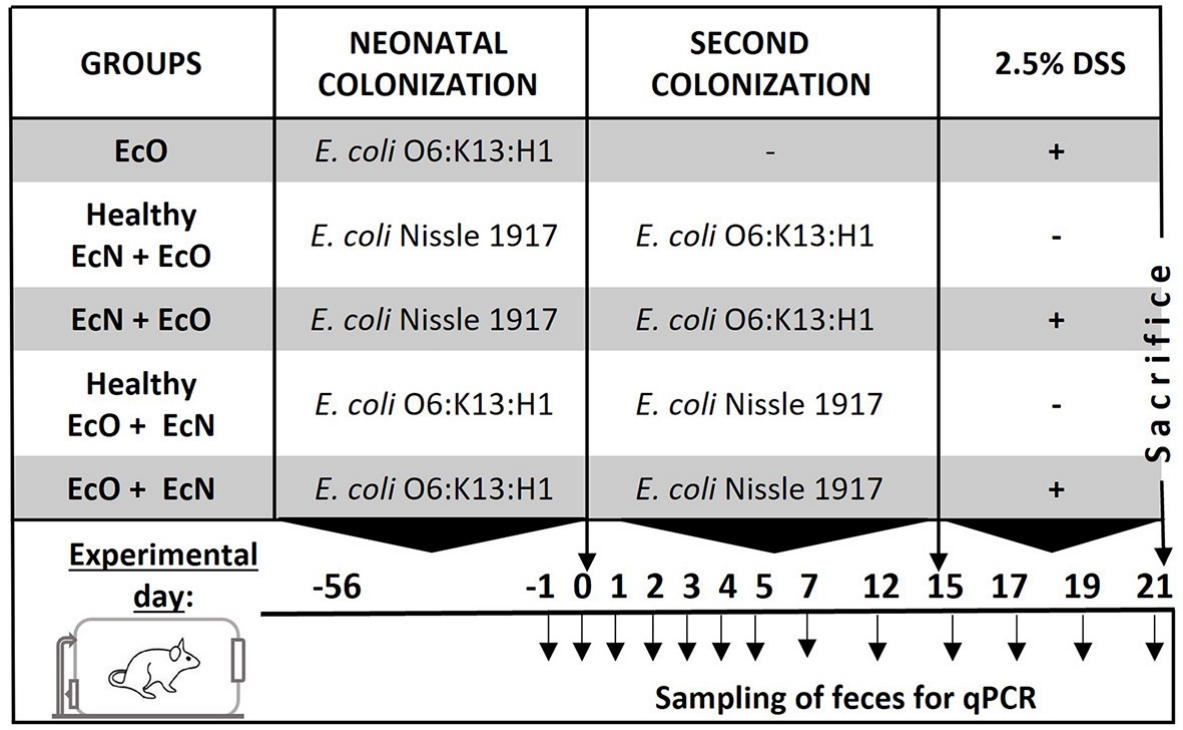
Experimental design. BALB/c mice neonatally colonized with *E. coli* Nissle 1917 or *E. coli* O6:K13:H1 were co-colonized by the second *E. coli* strain at the age of 2 months after birth. Fifteen days after the co-colonization with the second bacteria, intestinal inflammation was induced by 2.5% dextran sodium sulfate (DSS) administration in drinking water for 7 consecutive days. Mice colonized by both *E. coli* strains without DSS administration served as healthy controls. Fecal samples for qPCR detection of bacteria were collected throughout the whole experiment on the indicated days.

After 15 days of the second colonization, mice received autoclaved 2.5% w/v dextran sulfate sodium (DSS, M.W. 36–50 kDa; MP Biomedicals, Illkirch, France) in drinking water *ad libitum* for 7 consecutive days to induce acute colitis (see Figure 1, experimental design). Mice colonized by EcN+EcO or EcO + EcN without DSS-induced colitis served as healthy controls. The experiment was repeated three times. To ensure the efficiency of the 2.5% DSS solution, 8–10 weeks-old BALB/c mice reared in SPF conditions were used as a positive control for acute intestinal inflammation, where signs of inflammation such as rectal bleeding and weight loss were apparent (data not shown). At the end of the experiment, occult bleeding was evaluated in stool samples using the hemocult test (Hemocare, Care Diagnostic GmbH, Austria) and scored as follows:

- 0: No visible bleeding, hemocult negative.
- 1: No visible bleeding from anus, minor detection by hemocult.
- 2: Minor bleeding from anus, positive hemocult.
- 3: Large bleeding from anus, positive hemocult.

After sacrifice colon were aseptically removed, its length measured, and tissue segments fixed in Carnoy's fluid for 30 min, transferred into 96% ethanol, and embedded in paraffin.

### Histological evaluation of inflammation in the colon

Paraffin-embedded sections (5 μm) were cut and stained with hematoxylin and eosin (H&E) or Alcian Blue/Nuclear Fast Red (all from Vector Labs, Burlingame, USA) for mucin production. The samples were viewed under an Olympus BX 40 microscope equipped with an Olympus Camedia DP 70 digital camera, and the images were analyzed using Olympus DP-Soft. The degree of surface epithelium damage, crypt distortion, and mucin production in individual colon segments was evaluated, and the histopathological score was assessed according to Hudcovic et al. (2001).

### Immunohistochemical determination of zonula occludens (ZO)-1 in colon

Colon-descendent segments were embedded in O.C.T. Tissue-Tek (Sakura Finetek, Alphen aan den Rinj, Netherlands) and frozen in liquid nitrogen. Acetone-fixed cryosections (5 μm thick) were used for immunohistochemistry. Expression of ZO-1 was detected using rabbit anti-mouse polyclonal antibody (Zymed Laboratories, Carlsbad, CA, USA) and secondary antibody Cy3 goat anti-rabbit IgG (Biomeda, Foster City, USA). The samples were viewed under an Olympus BX 40 microscope equipped with an Olympus Camedia DP70 digital camera, and the images were analyzed using Olympus DP-Soft (Jourova et al., 2022).

### Detection of *E. coli* Nissle 1917 and *E. coli* O6:K13:H1 by qPCR

Fecal samples were collected 1 day before (day-1) intragastric administration of the second *E. coli* strain, 12 h after the administration of the second strain (day 0), and on subsequent days throughout the colonization of mice as depicted in the experimental design (Figure 1). Samples were stored at −40°C for qPCR analysis. A part of the fecal sample was serially diluted with sterile PBS and plated on modified McConkey agar supplemented with 2% raffinose, prepared according to Splichalova et al. (2014) (see Supplementary materials and methods and Supplementary Figure S2). Total DNA from the feces of colonized mice or pure *E. coli* Nissle 1917/*E. coli* O6:K13:H1 culture was isolated using the ZR Fecal DNA Kit according to the manufacturer‘s instructions (Zymo Research, USA). Agarose gel electrophoresis and UV spectrophotometry confirmed the purity, integrity, and concentration of nucleic acids.

To generate a standard curve for qPCR analysis, bacterial DNA was extracted from 150 μl of 10x diluted fecal samples in PBS (w/v) of monocolonized mice (EcN or EcO). Subsequently, the bacterial concentration (CFU/g of feces) corresponding to 1 μl of isolated DNA was determined by plating on LB agar (Himedia Laboratories, USA) and cultivating aerobically overnight at 37°C. A five-point standard curve (10-fold dilutions corresponding to 10^6^ to 10^2^ CFU per 1 μl of DNA) was used for absolute bacterial quantification of *E. coli* Nissle 1917 or O6:K13:H1 in qPCR reactions. The *C*_*T*_ values at the different dilution points were averaged, and the total number of bacterial cells was interpolated from the averaged standard curve as described elsewhere (Ott et al., 2004; Costa et al., 2022).

Amplification and detection were carried out in 96-well optical plates using the Bio-Rad Real-time PCR Detection System iQ5 (Bio-Rad laboratories, USA) in a final volume of 20 μl per reaction containing 1 μl of a 10x diluted sample of DNA, 2x MM Syto9 (Top-Bio, Czechia), a 0.4 μM concentration of each primer, and PCR water. For the detection of *E. coli* Nissle 1917, primers Muta-9/Muta-10 (forward: 5'gcg agg ta acct cga aca tg 3'; reverse: 5'cgg cgt atc gat aat tca cg 3') according to Blum-Oehler et al. (2003) were used. The quantitative PCR was performed with an initial hold of 95°C for 3 min, followed by 35 cycles of denaturation at 95°C for 45 s, annealing at 60°C for 45 s, and elongation at 72°C for 45 s, and with a final elongation step at 72°C for 10 min (Blum-Oehler et al., 2003).

For the amplification of *E. coli* O6:K13:H1, primers detecting the *hly*A gene were used (forward: 5'tca gga act ggt ttg aaa aa 3'; reverse: 5'att acc tgc agc tga aat ga 3') (Sheshko et al., 2006). The quantitative PCR was performed with an initial hold of 95°C for 5 min, followed by 35 cycles of denaturation at 95°C for 45 s, annealing at 56°C for 45 s, and elongation at 72°C for 45 s, with a final elongation step at 72°C for 10 min. After each qPCR run, melting curve analysis was performed to verify the presence of the desired amplicon.

### Colon fragment cultures

Fragments of the distal colon were washed with PBS to remove fecal contents, weighed, cut to approximately 100 mg each, and cultured in RPMI 1640 supplemented with 100 U/ml penicillin, 100 μg/mL streptomycin, 1% HEPES (all Sigma-Aldrich), and 10% heat-inactivated fetal calf serum at 37°C, a 5% CO_2_ for 48 h. Supernatants were collected and stored at −40°C until further processing (Hudcovic et al., 2012). The levels of cytokines IL-1β, IL-6, and TNF-α were determined using a Mouse Cytokine/Chemokine Multiplex Immunoassay (Millipore-Sigma, MO, USA) according to the manufacturer's instructions.

### Statistical analysis

Data are expressed as mean ± standard error of the mean (SEM). Data were analyzed using the Student's *t*-test or one-way ANOVA with Tukey's *post-hoc* test. All statistical analyses were conducted using GraphPad Prism 8.0 Software (San Diego, CA, USA).

## Results

### Analysis of adhesive and invasive properties of *E. coli* Nissle 1917 and *E. coli* O6:K13:H1

**Figure 2:**
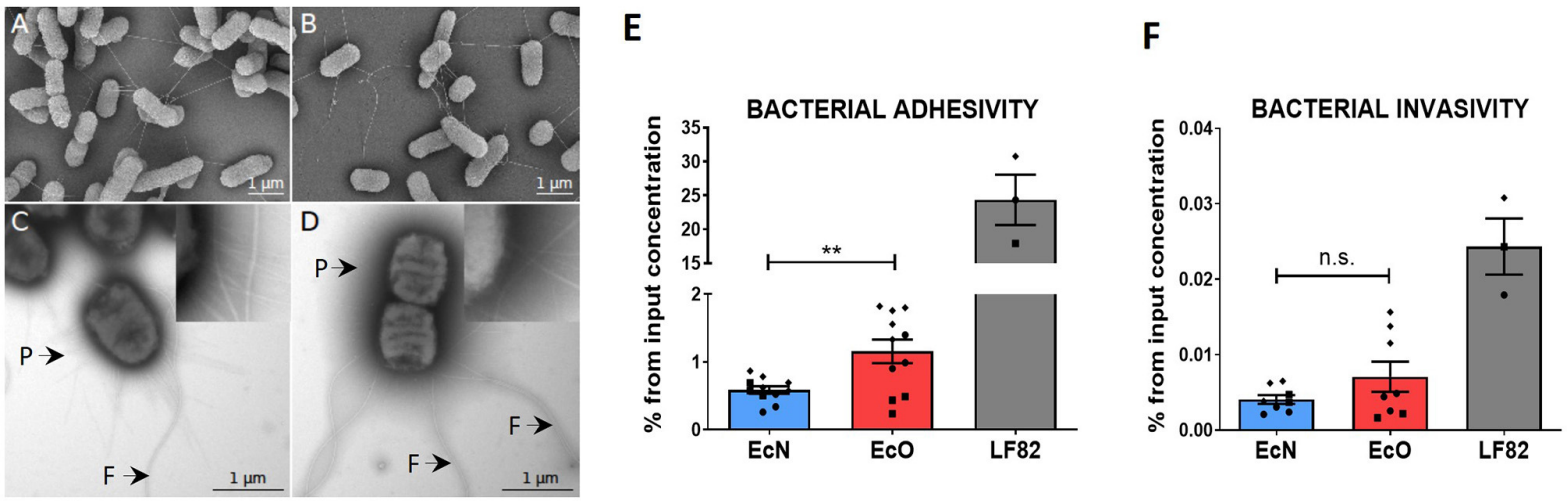
*In vitro* analysis of adhesive and invasive properties of *E. coli* Nissle 1917 and *E. coli* O6:K13:H1 by scanning and transmission electron microscopy and Caco-2 cell line transwell cultures. Scanning electron microscopy of **(A)** *E. coli* Nissle 1917 and **(B)** *E. coli* O6:K13:H1 shows a filamentous network via flagella. Scale bar = 1 μm. Transmission electron microscopy of negatively stained **(C)** *E. coli* Nissle 1917 and **(D)** *E. coli* O6:K13:H1 shows flagella (F) and pili (P) structures. Scale bar = 1 μm. Caco-2-polarized cell monolayers were cultured on transwell inserts for 21 days. Overnight bacterial cultures of *E. coli* Nissle 1917 (EcN), *E. coli* O6:K13:H1 (EcO), and *E. coli* LF82 (LF82) were added to the apical part of the insert and left to adhere/invade for 5 h. **(E)** Adherent bacteria were detected on the surface of intact Caco-2-polarized cells. **(F)** Invading bacteria were determined after antibiotic treatment and the removal of bacteria adhered to the surface of Caco-2 cells. Results are shown as % of adhered or invaded bacteria from bacterial input concentration and expressed as mean ± SEM. The data are pooled from three independent experiments with two to five biological replicates. Each experiment is denoted by a specific symbol (circle, square, and diamond). A Student's *t*-test was used for comparison between the EcN and EcO groups (**p < 0.01).

Scanning (Figures 2A, B) and transmission (Figures 2C, D, Supplementary Figures S1A, B) electron microscopy were performed on *E. coli* Nissle 1917 (EcN) and O6:K13:H1 (EcO) cultures to investigate the surface structures of bacterial cells. An overnight culture with elongated bacterial cells in a stationary growing phase was used for scanning electron microscopy analysis. For TEM analysis, a fresh culture of *E. coli* cells in the exponential phase was used to ensure the fine structures of both flagella and pili. Both strains exhibited regular rod-shaped structures with a mean cell length of approximately 1–2 μm and a mean thickness of approximately 0.8–1.2 μm (Supplementary Figure S1C).

Scanning electron microscopy of EcN (Figure 2A) and EcO (Figure 2B) displayed a formed filamentous network with flagella connecting bacterial cells. The transmission electron microscopy analysis (Figures 2C, D, Supplementary Figures S1A, B) confirmed the presence of typical filamentous structures on the surface of strains, including flagella and pili. The long and thicker flagella had an average diameter of 17 ± 2 nm (Supplementary Figure S1D), and the thinner and shorter pili had diameters smaller than 10 nm. The presence of pilli and flagella structures was found on both EcN and EcO, ensuring their potential for adhesion to the epithelial cells in the host or cell cultures.

The ability of EcN and EcO to adhere to and invade epithelial cells was tested using a polarized Caco-2 transwell culture, which mimics the intestinal environment. The adherent/invasive strain *Escherichia coli* LF82, originally isolated from the ileum of a patient with Crohn's disease and capable of disrupting the apical junctional complexes in polarized epithelia, was used as the positive control (Boudeau et al., 2003; Wine et al., 2009). As expected, *E. coli* LF82 showed high adhesive and invasive ability in the polarized Caco-2 cell line.

Both analyzed EcN and EcO strains displayed weaker efficiency in adhering to (Figure 2E) and invading epithelial cells compared to the positive control (Figure 2F). Nonetheless, EcO was significantly more adhesive and showed a trend toward higher invasiveness than EcN. These results suggest that EcO, as a uropathogenic strain, has better mechanisms for attaching to the epithelial cells of the host.

### Stimulation of human embryonic kidney 293 cells stably transfected with TLR4, TLR2/CD14- and NOD2 by *E. coli* Nissle 1917 and *E. coli* O6:K13:H1

**Figure 3:**
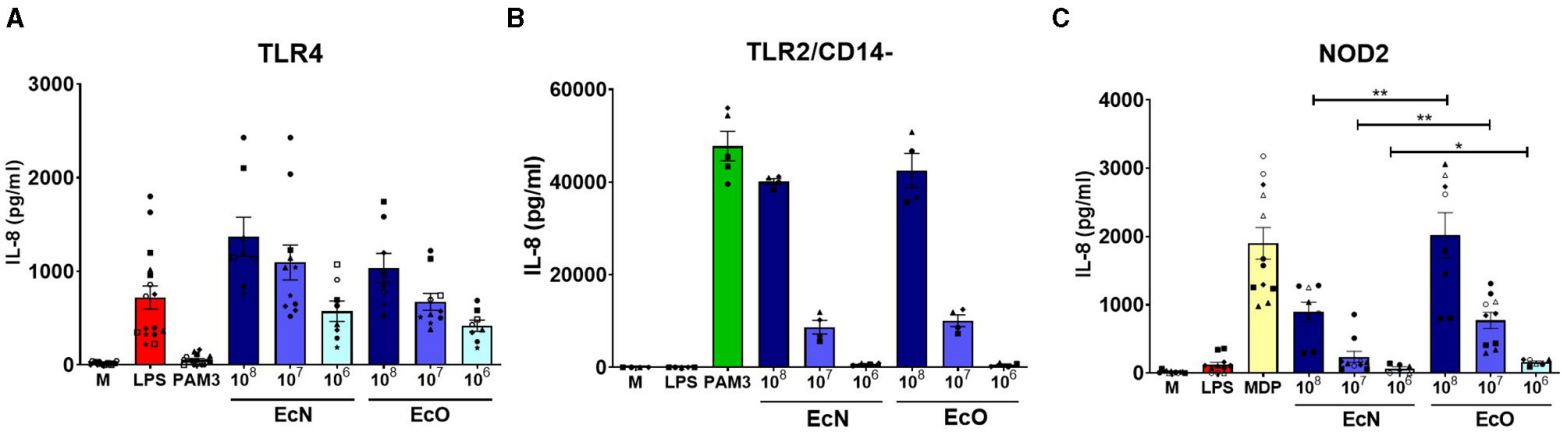
Stimulation of TLR4, TLR2/CD14-, and NOD2 receptors by *E. coli* Nissle 1917 and *E. coli* O6:K13:H1. Human embryonic kidney cells (HEK 293) stably transfected with an expression vector for **(A)** human TLR4 (293-hTLR4/MD2/CD14), **(B)** human TLR2 (293-hTLR2/CD14-), or **(C)** human NOD2 (293-hNOD2) were stimulated with formalin-inactivated *E. coli* Nissle 1917 (EcN) or *E. coli* O6:K13:H1 (EcO) for 20 h. Stimulation was performed at concentrations of 10^6^, 10^7^, or 10^8^ CFU/ml. Cells stimulated with lipopolysaccharide (LPS-EB, 1 μg/ml), PAM3CSK4 (PAM3, 1 μg/ml), or muramyl dipeptide (MDP, 1 μg/ml) were used as positive controls. Untreated cells (M) were used as negative controls. The data are pooled from eight experiments performed for TLR4 analysis, four experiments for TLR2/CD14-, and six experiments for NOD2 analysis and expressed as the production of IL-8 (pg/ml). Each experiment is denoted by a specific symbol with one or two biological replicates for each bacterial concentration. Differences between EcN and EcO stimulation were statistically evaluated using the *t*-test (**p < 0.01 and *p < 0.05).

HEK 293 cells stably transfected with human genes for pattern recognition receptors TLR4, TLR2/CD14- or NOD2 were used to investigate the innate signaling pathways activated by both *E. coli* strains. Our results showed that both strains are strongly recognized by TLR4 and TLR2/CD14- receptors in a similar dose-dependent manner (Figures 3A, B). There were no differences in signaling through the TLR4 or TLR2/CD14- pathways between the studied strains at the tested bacteria concentrations, suggesting that surface structures such as LPS or lipoproteins are sensed similarly. However, EcO was recognized more intensively by the NOD2 receptor than EcN, suggesting differences in the sensing of peptidoglycan structures (Figure 3C).

### Strains *E. coli* Nissle and O6:K13:H1 have similar ability to activate dendritic cells but induce distinct cytokine response *in vitro*

**Figure 4:**
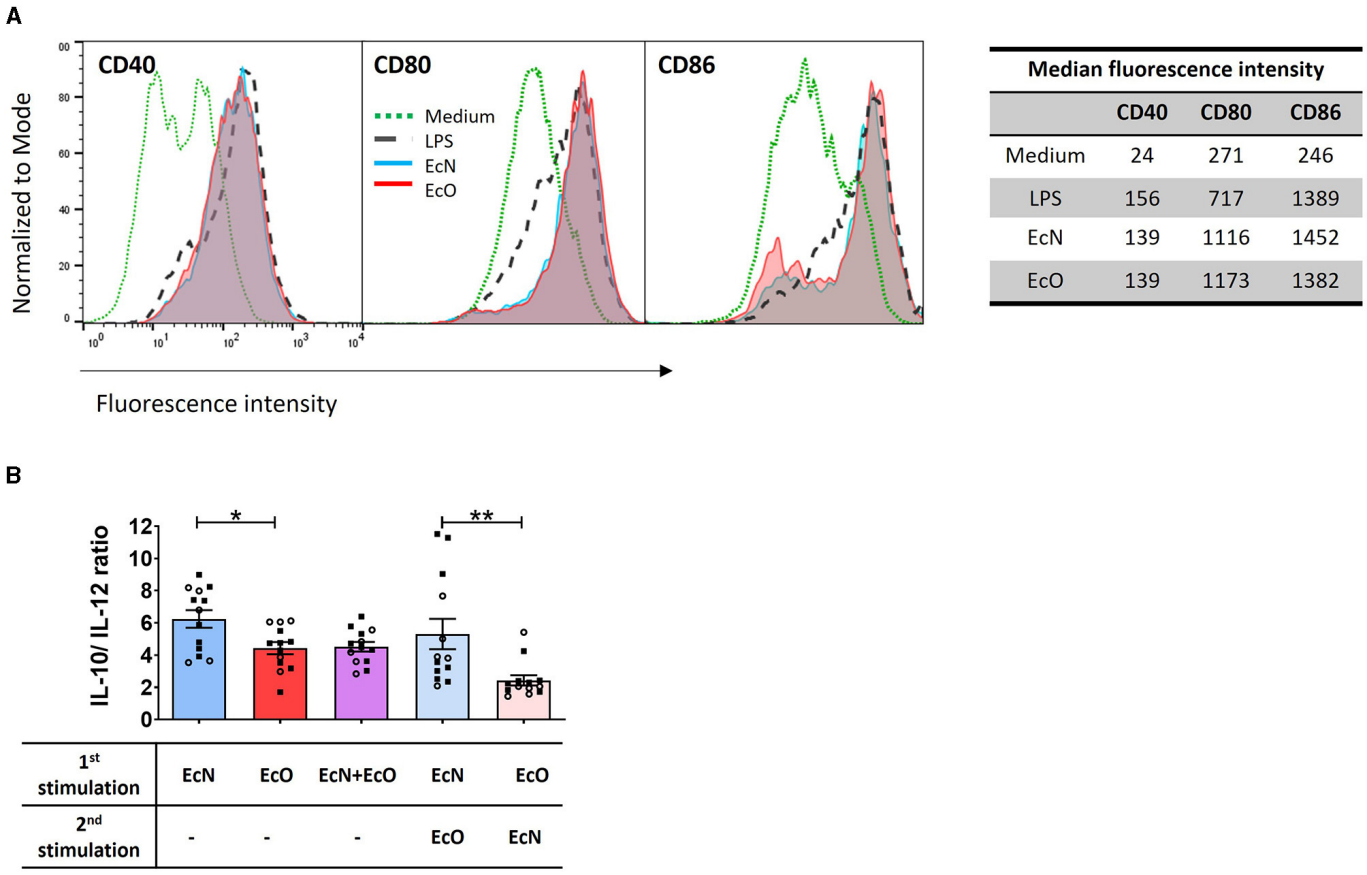
*E. coli* Nissle 1917 and *E. coli* O6:K13:H1 strains activate the maturation and cytokine production of BM-DCs. **(A)** BM-DCs from naïve mice were cultured with formalin-inactivated *E. coli* Nissle 1917 (EcN) and *E. coli* O6:K13:H1 (EcO) for 18 h. Lipopolysaccharide from *E. coli* (LPS-EB, 1 μg/ml) was used as a positive control. Untreated cells (medium) served as a negative control. The expression of CD40, CD80, and CD86 was assessed using flow cytometry. BM-DCs were gated as MHCII^+^CD11c^+^. Numbers represent the median fluorescence intensity from one representative experiment out of two. **(B)** BM-DCs were stimulated with formalin-inactivated strains EcN, EcO, and a mixture of EcN and EcO or pre-incubated first with one strain (EcN or EcO, resp.) for 5 h, and after the washing step, the cells were stimulated by the second strain (EcO or EcN, resp.). The cytokines IL-10 and IL-12p70 were determined in cell culture supernatants by ELISA. The results are expressed as the ratio of IL-10/lL-12p70 production. The data are pooled from two independent experiments with a total of 13 biological replicates and expressed as the mean ± SEM. A statistical evaluation by *t*-test was performed for differences analysis between EcN and EcO stimulation or cultures sequentially stimulated by both EcN and EcO strains (*p < 0.05 and **p < 0.01).

BM-DCs derived from naïve BALB/c mice were used as an *in vitro* model to investigate the immunostimulatory potential of both *E. coli* strains on immune antigen-presenting cells. The expression of co-stimulatory markers CD40, CD80, and CD86 was assessed to determine the activation status of BM-DCs after stimulation with each bacterial strain. These surface markers were highly induced by both strains, comparable to the positive LPS control (Figure 4A).

BM-DC cultures were stimulated by *E. coli* strains separately (EcN or EcO), in a mixture of both strains (EcN+EcO), or sequentially pre-incubated with the first strain (EcN or EcO) and after washing, stimulated with the second strain (EcO or EcN). The levels of IL-10 and IL-12p70 were measured in the BM-DCs supernatants (Figure 4B). The data show that stimulation of BM-DCs with the EcN strain alone induces a higher anti-inflammatory cytokine ratio of IL-10/IL-12p70 compared to the EcO strain. This anti-inflammatory effect was maintained even when BM-DCs were pre-incubated with EcN and then stimulated with EcO. However, this effect was not apparent in BM-DC co-cultures with the EcN+EcO mixture or when EcN was added as the second stimulus after EcO (Figure 4B). These results indicate that EcN induces an anti-inflammatory response in immune cells that persists even after subsequent exposure to EcO. Conversely, EcN added as the second strain cannot alter the immune response pre-induced by the pathogenic EcO strain.

### Neonatal colonization by *E. coli* Nissle 1917 ameliorates DSS-induced colitis promoted by *E. coli* O6:K13:H1 strain

To investigate the effect of priority colonization on the host, we used an *in vivo* mouse model of acute ulcerative colitis induced by administering 2.5% DSS in drinking water. Mice neonatally colonized by EcN received one dose of EcO (1.1 × 10^8^ CFU) intragastrically 15 days prior to colitis induction (EcN+EcO group). To test the anti-inflammatory potential of EcN in neonatally EcO monocolonized mice, one dose of EcN (1.91 × 10^8^ CFU) was administered intragastrically as a second strain 15 days before the induction of DSS-colitis (EcO+EcN group).

**Figure 5:**
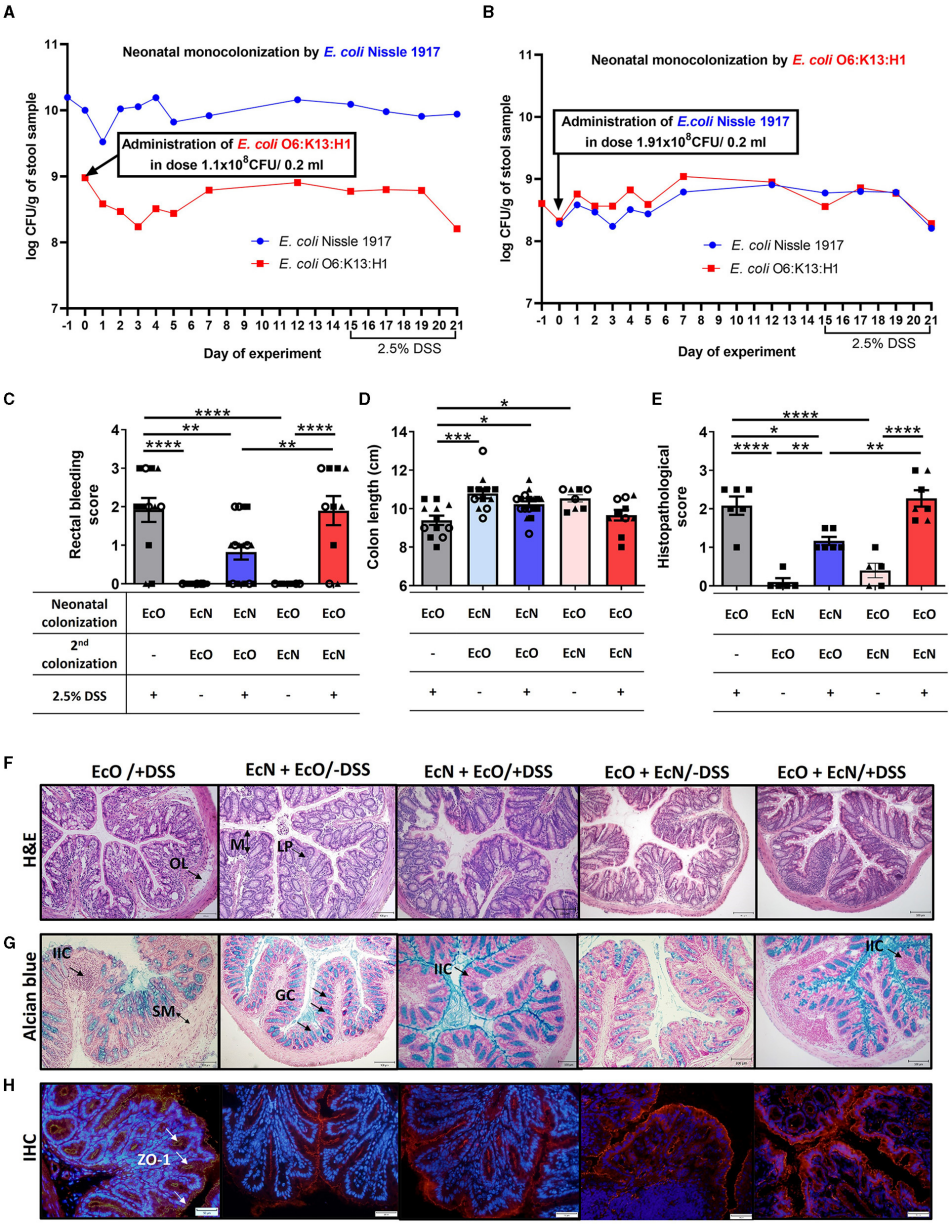
Neonatal colonization but not one-dose treatment by *E. coli* Nissle 1917 protects against the development of severe colitis in an experimental mouse model. The efficiency of neonatal and second re-colonization by *E. coli* Nissle 1917 (EcN) and *E. coli* O6:K13:H1 (EcO) was detected by qPCR from fecal samples throughout the experiment. **(A)** Neonatally colonized mice by EcN with one-dose treatment by EcO reached stable, high colonization efficiency of their parenteral *E. coli* strain. **(B)** *E. coli* Nissle 1917 was administered in one dose as the second strain to EcO-colonized mice. Stable colonization efficiency was detected by qPCR throughout the whole experiment, using fecal samples. **(C)** The rectal bleeding score was analyzed in mice neonatally colonized by EcN or EcO, treated with the second strain (EcO or EcN), and with 2.5% DSS to induce acute ulcerative colitis. **(D)** Shortening of the colon was evaluated in experimental mice on sacrifice day. **(E)** The histopathological score was determined in colon tissue sections in Carnoy fixed and paraffin-embedded sections. **(F)** Representative colon descendens cross-sections stained by hematoxylin and eosin (H&E; magnification 200x, scale 100 μm) (LP, lamina propria; OL, oedematic loci; M, mucosa). **(G)** Colon descendens tissue cross-sections stained by alcian blue and nuclear fast red are shown for visualization of mucus production (magnification 200x, scale 100 μm) (IIC, inflammatory immune cells; SM, submucosa; GC, goblet cells). **(H)** Immunohistochemical staining (IHC) of zonula occludens-1 in colon tissue cryosections (magnification 400x, scale 50 μm). Data are expressed as one representative out of three independent experiments **(A, B, E)** or as the mean ± SEM from three pooled independent experiments **(C, D)**. Each experiment is denoted by a specific symbol (square, triangle, and circle). A one-way ANOVA with Tukey's post-test was used for the determination of statistical differences (*p < 0.05, **p < 0.01, ***p < 0.001, and ****p < 0.0001).

The colonization efficiency of both *E. coli* strains was analyzed in fecal samples by qPCR or bacteria cultivation on modified McConkey agar supplemented with 2% raffinose on indicated days (see experimental design Figure 1, Supplementary Figure S2). We found that the concentration of EcN in neonatally colonized mice reached up to 10^10^ CFU/g of feces (Figure 5A, Supplementary Figure S2C) in comparison to EcO neonatal colonization, which reached 10^8^ CFU/g of feces (Figure 5B, Supplementary Figure S2D). The concentrations of EcO bacteria remained stable in both experiment setups, regardless of whether the EcO strain was the first (neonatally colonized) or the second strain in EcN-colonized mice.

Conversely, EcN administered as the second strain did not reach as high a concentration as in the neonatal setup, maintaining a level of 10^8^ CFU/g of feces (Figure 5B, Supplementary Figure S2D).

Further, we investigated the priority colonization effect by colonizing germ-free mice at 6 weeks of age with either individual EcN or EcO strains or with a 1:1 mixture of EcN and EcO (Supplementary Figure S3A). Mice mono-colonized with individual EcN and EcO strains exhibited stable high colonization levels, reaching 10^9^-10^10^ CFUs per gram of feces from the second day of the experiment. We detected a high concentration of the EcO strain in monocolonized mice, but in mice colonized by a mixture of EcN+O, the concentration of EcO was 10 times lower. Furthermore, the concentration of EcN strain in feces reached nearly the same level in mice mono-colonized with the individual strain, bi-colonized with an EcN+O mixture (Supplementary Figure S3B), or EcN-neonatally colonized mice with EcO as the second strain (Figure 5A, Supplementary Figure S2C). Despite the stable coexistence of both *E. coli* strains in the mouse intestine, the EcN strain was able to outcompete the abundance of EcO by a competitive interaction, showing a 100-fold reduction in EcO abundance in neonatally pre-colonized EcN mice or a tenfold reduction in mice colonized simultaneously with an EcN+O mixture.

Disease progression in experimental mice was characterized by rectal bleeding, the shortening of the colon length, and histological evaluation after DSS treatment (Figures 5C–E, Supplementary Figures S3C–E). The histopathological scoring was based on the infiltration of inflammatory cells into the lamina propria and submucosa, submucosa thickening, and the loss of the entire crypt with retained surface epithelium (Hudcovic et al., 2001). Rectal bleeding and the shortening of colon length were most pronounced in mice monocolonized with EcO strain alone (regardless of the colonization of newborn or adult mice) and treated with 2.5% DSS (EcO+DSS groups) compared to healthy control (EcN+EcO or EcO+EcN without DSS) (Figures 5C, D, Supplementary Figures S3C–E).

Histopathological findings included infiltration of inflammatory cells (IIC) into the lamina propria (LP), the thickening of submucosa (SM), the appearance of oedematic loci (OL) in the mucosa (M) structure, and the disappearance of the goblet cells (GC) from the lamina propria in the EcO+DSS group of mice (Figures 5E–G, Supplementary Figures S3F–H). As previously reported for neonatally colonized mice (Hudcovic et al., 2007), adult mice colonized with the EcN strain and treated with DSS exhibited negligible signs of inflammation and histological characteristics comparable to healthy controls, suggesting its anti-inflammatory potential (Supplementary Figures S3C–H).

Similarly, mice that were neonatally colonized with EcN and then administered the pathobiont EcO as a second strain showed lower occurrence of rectal bleeding after DSS treatment and the preservation of colon length similar to healthy controls (Figures 5C, D). Histopathological analysis revealed only negligible changes in colonic mucosa, characterized by small infiltration of cells into lamina propria, preserved structure of villi and crypts with an undisturbed epithelial layer, and mucus production ability (Figures 5E–G). Conversely, the mice treated with EcN as the second colonizing bacteria (EcO+EcN+DSS) showed bleeding scores and colon shortening similar to EcO-monocolonized mice after DSS treatment. Histological analysis of the colon mucosa confirmed macroscopic signs of inflammation, indicating that one-dose treatment with EcN is ineffective in preventing DSS-induced intestinal inflammation in EcO-colonized mice. Similar results were observed in adult GF mice colonized by a mixture of EcN and EcO (Supplementary Figures S3C–H), showing moderate signs of inflammation, rectal bleeding, colon shortening, and mild histopathological changes in the colonic mucosa.

Altered intestinal barrier function through decreased expression of tight junction proteins, such as ZO-1, is a critical factor in the development of intestinal inflammation in mouse models of colitis or human IBD. In this study, we investigated whether neonatal EcN colonization or one-dose EcN treatment influenced the alteration of ZO-1 induced by DSS treatment in EcO-colonized mice. Immunohistochemistry staining (IHC) revealed reduced ZO-1 expression after DSS administration in the mice colonized with only EcO, compared to healthy controls. In contrast, preserved ZO-1 expression was evident in both neonatally EcN-colonized and one-dose EcN-treated mice (Figure 5H).

These results demonstrate the priority effect of EcN on the host, showing that EcN neonatal colonization prior to EcO colonization protects the host from the induction of the inflammatory response driven by the pathogenic EcO strain.

### Neonatal colonization by *E. coli* Nissle 1917 downregulated the level of proinflammatory cytokines in colon tissue

**Figure 6:**
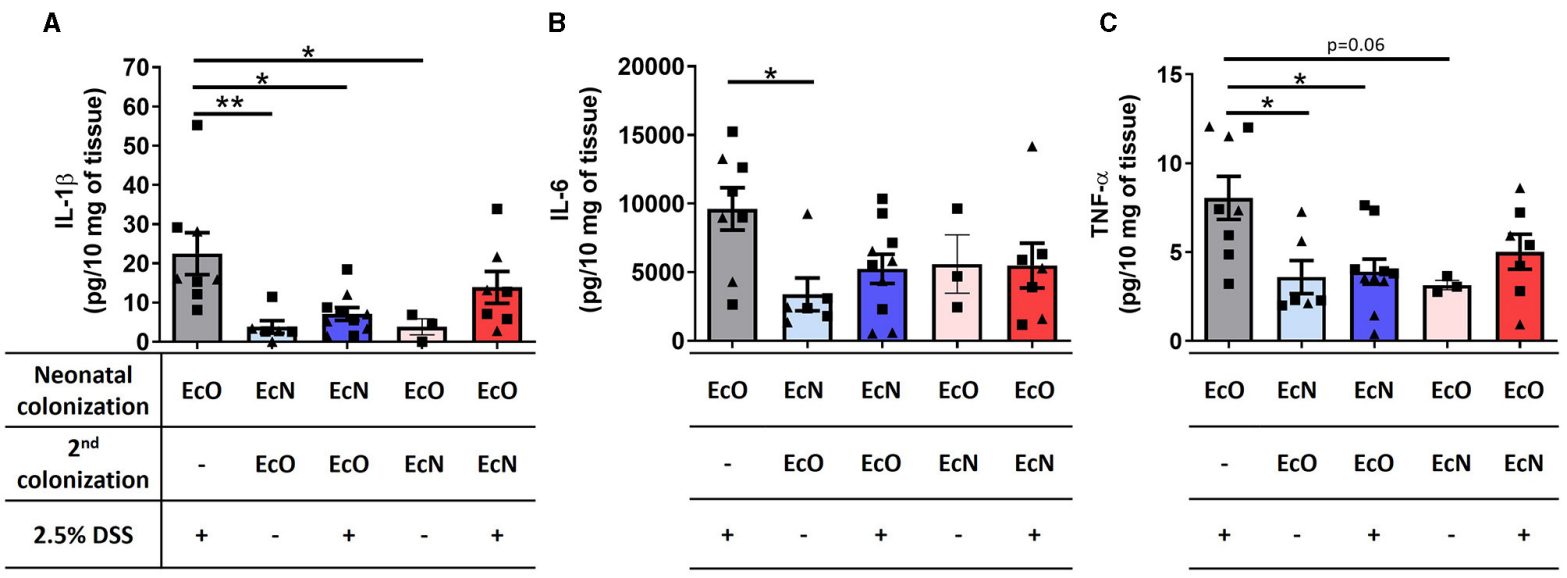
Neonatal colonization by *E. coli* Nissle 1917 downregulates the level of proinflammatory cytokines in colon tissue. **(A)** IL-1β, **(B)** IL-6, and **(C)** TNF-α were analyzed in the supernatants of colon tissue fragment cultures of mice neonatally colonized by *E. coli* Nissle 1917 (EcN) or by *E. coli* O6:K13:H1 (EcO), treated with the second strain (EcO or EcN), and with/without 2.5% DSS to induce acute ulcerative colitis. The data are pooled and expressed as the mean ± SEM of two independent experiments. Each experiment is denoted by a specific symbol (square and triangle). A one-way ANOVA with Tukey's post-test was used for the determination of statistical differences (*p < 0.05 and **p < 0.01).

Changes in the cytokine microenvironment in the gut were analyzed in the supernatants of colon tissue fragment cultures from the mice neonatally colonized by EcN or EcO, co-colonized by EcO or EcN, and treated with 2.5% DSS to induce intestinal inflammation. Similar to macroscopic and histopathological evaluation, the mice monocolonized by EcO and treated by DSS showed a significant upregulation of proinflammatory cytokines IL-1β (Figure 6A), IL-6 (Figure 6B), and TNF-α (Figure 6C) compared to healthy controls without DSS treatment. Notably, we found that neonatal colonization by EcN efficiently protected against the upregulation of these proinflammatory cytokines in DSS-treated mice, even when the potentially pathogenic EcO was administered 2 weeks before colitis induction by DSS. Insignificant downregulation of these cytokines was also observed in mice where EcN was administered as the second colonizing stimulus in one dose only. These results highlight the potential of *E. coli* Nissle 1917 to protect against inflammation, even in the presence of the potentially pathogenic *E. coli* O6:K13:H1 strain.

## Discussion

The first year of life is crucial for newborns to develop their commensal microbiota, which influences the development of their immune system and has corresponding consequences for their lifelong health. The neonatal gut is an aerobic environment at birth, where only facultative anaerobes such as *Escherichia coli* and *Enterococcus* eventually create a favorable anaerobic environment for colonization by subsequent microbes (Jost et al., 2012; Matamoros et al., 2013). This pioneering microbiome has lifelong effects and shows that colonization in childhood can persist into adulthood, influencing the development of many diseases. These findings suggest that the sequence and type of bacteria that colonize our bodies after birth are of utmost importance for our future health (Faith et al., 2013).

In this study, we compared two related strains of *E. coli* of serogroup O6: probiotic *E. coli* Nissle 1917 and uropathogenic *E. coli* O6:K13:H1, focusing on their ability for neonatal colonization and competitive interaction in the intestinal mucosa, their influence on the development of the immune system, and their effects on the development of intestinal inflammation in adult mice.

Using *in vitro* experiments, we found that both strains possess distinct properties regarding adhesiveness to Caco-2 epithelial cells and modulation of the Th1 immune response in BM-DC cultures. In gnotobiotic *in vivo* experiments, we demonstrated that neonatal colonization with *E. coli* Nissle 1917 confers colonization resistance to *E. coli* O6:K13:H1 and protects mice from developing experimentally induced intestinal inflammation.

Conversely, in mice neonatally colonized with the pathobiont *E. coli* O6:K13:H1, one-dose administration of *E. coli* Nissle 1917 does not reach as high a concentration in the intestine as in the neonatal setup and fails to protect the mice from developing acute ulcerative colitis. These findings highlight the critical importance of the order of arrival of the intestinal colonizing strains.

Finally, we showed that the colonization of adult germ-free mice with a mixture of EcN and EcO strains in a 1:1 ratio (Supplementary Figure S3) had only a weak effect on the alleviation of DSS-induced intestinal inflammation compared to the mice neonatally pre-colonized with EcN strain, where EcO was the second colonizing strain or mice that were mono-colonized only with the EcN strain throughout the entire experiment. These results demonstrate the priority effect of neonatal colonization with *E. coli* Nissle on the host's immune response to inflammatory stimuli later in life.

Studies in IBD patients have found an increased incidence of *E. coli* strains that can adhere to and invade epithelial cells *in vitro* (Boudeau et al., 1999). This type of adherent-invasive *E. coli* strain (also known as AIEC) has specific factors for adherence to intestinal epithelial cells, invasion into the cytoplasm of the infected eukaryotic cells, and intracellular replication in epithelial cells and macrophages, which consequently facilitates the development of IBD in humans (Barnich and Darfeuille-Michaud, 2007).

The impact of neonatal colonization by the LF82 strain (belonging to the AIEC group) together with altered Schaedler microbiota (ASF) in comparison to colonization by LF82 in adult ASF mice was introduced by Wymore Brand et al. (2022). The authors showed that neonatal colonization with LF82 aggravated DSS-induced colitis to a greater extent than when the strain was administered to adult mice. Their results are in line with our study, highlighting the importance of the timing of bacterial strain arrival and its consequences on the host's inflammatory response.

For scientists, it is crucial to search for potentially pathogenic *E. coli* isolates with properties similar to those possessed by the AIEC strains, as these could be associated with the pathogenesis of IBD. In this context, Martinez-Medina et al. (2009) showed that the uropathogenic strain *E. coli* OL96a can adhere to and invade the intestinal epithelial cells, shares some virulence genes with AIEC, and is phylogenetically related to intestinal AIEC strains. Similarly, in this study, we investigated *E. coli* O6:K13:H1 as an extraintestinal strain for its ability to adhere to and invade epithelial cells, modulate immune responses, and induce intestinal inflammation using *in vitro* and *in vivo* experiments.

Many pathogenic bacteria bind to the host tissue via adhesive surface organelles called pili, which are important virulence factors for various diseases, especially infections of the urinary, genital, and gastrointestinal tracts. In addition, type 1 pili have been found to be responsible for bacterial invasion and persistence in target cells (Capitani et al., 2006). Using transmission and scanning electron microscopy, we showed that both EcO and EcN strains express sufficient numbers of pili and flagella structures, suggesting their potential for adhesion and invasion into host epithelial cells.

Using transwell cultures, we demonstrated that EcO adheres more strongly to the Caco-2 epithelial cell line than the probiotic EcN strain but approximately 20 times less than the AIEC control strain LF82.

Furthermore, we found that the EcO strain has no invasive activity, indicating that this strain does not belong to the AIEC group. These results are consistent with the fact that uropathogenic *E. coli* strains should possess the factors that enable better adhesion to epithelial cells, quorum sensing, and biofilm formation, which directly or indirectly contribute to the development of urinary tract infections (Shah et al., 2019).

Epithelial and immune cells can recognize LPS, flagella, and peptidoglycan of *E. coli* via pattern recognition receptors (PRR). The interaction between TLRs and *E. coli* cell structures could activate the signaling pathways leading to the production of proinflammatory cytokines such as TNF-α or IL-6. Using HEK 293 cells, we demonstrated that both EcN and EcO strains are strongly recognized by TLR4 and TLR2 receptors in a similar dose-dependent manner. However, EcO was more intensely recognized by NOD2 receptors than EcN, suggesting differences in recognizing peptidoglycan structures. NOD2 variants are associated with Crohn's disease, in which a defect in recognizing commensal bacteria leads to gastrointestinal inflammation (Al Nabhani et al., 2017). An overexpression of NOD2 in Caco-2 cells led to a greater increase in the autophagy process, which was associated with decreased survival of AIEC and the release of proinflammatory cytokines (Negroni et al., 2016). These results highlight the important role of NOD2 signaling in activating autophagy processes as a host defense mechanism to control the overgrowth of pathogenic bacteria (Al Nabhani et al., 2017).

Cooney et al. (2010) have demonstrated that NOD2 signaling is crucial for muramyl dipeptide-induced autophagy in DCs, as well as for subsequent MHC class II upregulation, DC activation, and antigen presentation. In this study, we compared EcN and EcO strains in terms of their ability to activate BM-DCs. Interestingly, the expression of CD40, CD80, and CD86 co-stimulatory surface markers was induced similarly by both strains and was comparable to the LPS control. However, we found that stimulation of BM-DCs with the EcN strain itself induces a higher anti-inflammatory cytokine ratio (IL-10/IL-12p70) than stimulation with the EcO strain. This phenomenon was preserved even when the BM-DC culture was pre-incubated by EcN before adding strain EcO to the culture.

Conversely, this effect was not apparent in BM-DC cultures co-incubated with a mixture of EcN and EcO, nor when EcN was added as the second bacterial stimulus. Our results are in line with the findings by Güttsches et al. (2012), showing that EcN lysate and its LPS induced the same level of IL-12p40 in comparison to the uropathogenic *E. coli* W536 strain but upregulated IL-10 production in human PBMC cultures.

Boudeau et al. (2003) demonstrated a significant inhibitory effect of *E. coli* Nissle 1917 on the adhesion and invasion of AIEC in intestinal epithelial cell lines. Similarly, Leatham et al. (2009) showed that *E. coli* Nissle 1917 limits the growth of pathogenic *E. coli* O157:H7 in the intestine of streptomycin-treated mice. In agreement with these results, this study shows that neonatal colonization of germ-free mice with EcN and subsequent administration of uropathogenic EcO allowed the growth of the EcO strain to a lower but stable level compared to the levels of the first colonizing EcN strain. Conversely, the concentration of the EcO strain in neonatally colonized mice never reached the levels of the EcN strain, suggesting an adaptation of the EcO strain to an extraintestinal niche.

The finding that EcO levels remain stable in neonatally colonized mice after the administration of the EcN strain suggests that the effect of EcN is not mediated by competitive exclusion of the pathogenic strain. Despite the stable coexistence of both *E. coli* strains in the intestinal environment of the mice, we demonstrated a competitive interaction that favors the early colonizing EcN strain over the later colonizing EcO strain. This so-called priority effect was described by Segura Munoz et al. (2022) for strains of *Akkermansia muciniphila* and *Bacteroides vulgatus*, in which the competitive ability of the strain has been influenced by the time of arrival.

Of particular importance is our demonstration that the primary effect of the EcN strain implies its anti-inflammatory potential for the host. We demonstrated that neonatal colonization with *E. coli* Nissle 1917 inhibited the contribution of EcO infection to intestinal inflammation upon DSS treatment.

Conversely, a single treatment with EcN is ineffective in protecting the development of DSS-induced ulcerative colitis in mice neonatally colonized with pathobiont EcO.

Our results are in line with the study by Pradhan and Weiss (2020), who investigated the probiotic properties of the EcN strain in human intestinal organoids (HIOs). They showed that pre-incubation of HIOs with EcN before they were challenged with EHEC or UPEC prevented the loss of epithelial barrier function, loss of E-cadherin expression, and increased production of reactive oxygen species and apoptosis. They described that EcN did not replicate in HIO co-culture like the pathogenic strains, thus conferring protection via activation of host defenses rather than eliminating competing strains.

Overall, we have described the interaction between two *E. coli* strains of the same O6 serotype, the uropathogenic O6:K13:H1 and the probiotic Nissle 1917 (O6:K5:H1), with regard to their effects on the host. Given the importance of the first colonizing bacterial strains in developing the immune response, we investigated how the order of arrival of these *E. coli* strains influences the host's response to inflammatory stimuli and the long-term effects on health.

Our results demonstrate that *E. coli* Nissle 1917 has the potential to protect against DSS-induced intestinal inflammation, even after co-infection with *E. coli* strain O6:K13:H1, which has proinflammatory potential. This study emphasizes the importance of a probiotic intervention approach, particularly with the *E. coli* Nissle strain, during the perinatal/neonatal period to prevent an aberrant microbiome and dysbiosis-related diseases such as IBD in an individual's later life.

Future research should explore additional strains with known niche properties, assess their competition within the broader microbial community, and focus on the precise mechanisms by which strains coexist, compete, and impact host health.

## Data availability statement

The raw data supporting the conclusions of this article will be made available by the authors, without undue reservation.

## Ethics statement

The animal study was approved by the Committee for Protection and Use of Experimental Animals of the Institute of Microbiology of the Czech Academy of Science. The study was conducted in accordance with the local legislation and institutional requirements.

## Author contributions

TH: Project administration, Writing – review & editing, Writing – original draft, Supervision, Methodology, Investigation. PPH: Writing – original draft, Investigation, Formal analysis, Data curation. HK: Writing – review & editing, Conceptualization. OB: Writing – review & editing, Methodology, Formal analysis, Data curation. OK: Writing – review & editing, Methodology, Formal analysis. MS: Writing – review & editing, Conceptualization. DS: Writing – review & editing, Writing – original draft, Visualization, Investigation, Formal analysis, Data curation.
