# Supplementary material for: Priority order of neonatal colonization by a probiotic or pathogenic Escherichia coli strain dictates the host response to experimental colitis
Source: Front Microbiol. 2024 Aug 14;15:1393732. doi: 10.3389/fmicb.2024.1393732 (PMC11349737; doi:10.3389/fmicb.2024.1393732)
Supplement: Supplementary file 1 [file Data_Sheet_1.pdf]

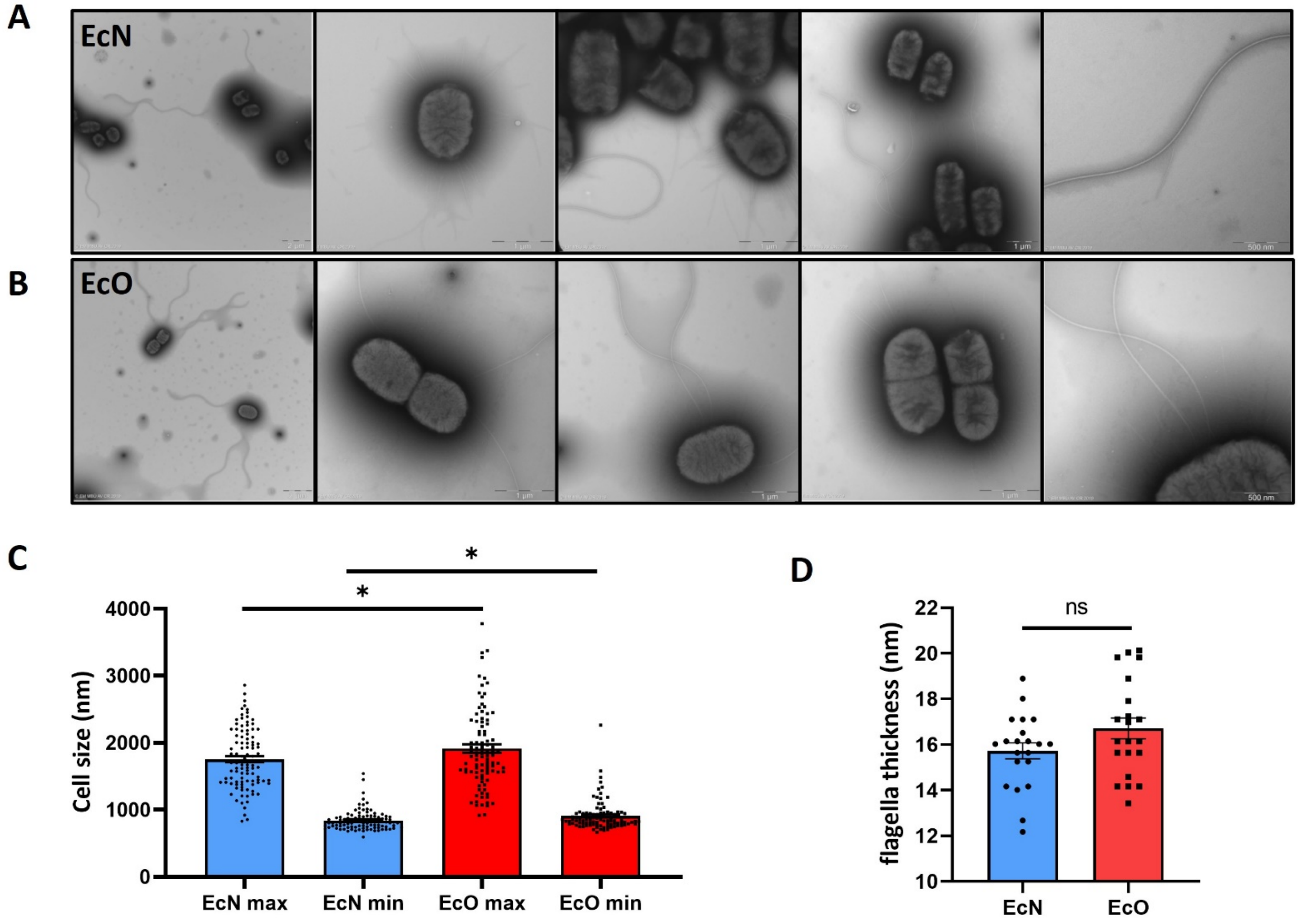

Figure S1: Visualization and quantification of cellular morphology of *E. coli* Nissle (EcN) and *E. coli* O6:K13:H1 (EcO) strains. Representative Transmission Electron Microscopy (TEM) pictures of **(A)** EcN and **(B)** EcO strains with flagella and pili structures on their surface (depicted scale 2 µM, 1 µM or 500 nM). **(C)** Analysis of bacterial cell length (EcN max, EcO max) or width (EcN min, EcO min) from Scanning Electron Microscopy (SEM) images were performed in 100 EcN or EcO cells using the Particle Analysis module of the electron microscopy processing software AnalySis 5.2. **(D)** The flagella thickness was determined in TEM images using the Particle Analysis module of the electron microscopy processing software AnalySis 5.2. Differences between EcN and EcO cell size were statistically evaluated by *t*-test (*$p < 0.05$).

A

	EcN	EcO		EcN	EcO		EcN	EcO
Malonate	-	-	Mannitol	+	+	Glucose	+	+
Simmons citrate	-	-	Trehalose	+	+	Dulcitol	-	-
Arginine	+	-	Sucrose	-	-	Raffinose	+	-
Urease	-	-	Cellobiose	-	-	Melibiose	+	+
Ornithine	+	+	Adonitol	-	-	Rhamnose	+	+
Lysine	+	+	Inositol	-	-	Sorbitol	+	+
Hydrogen sulfide	-	-	β-galactosidase	+	+	Aesculine	-	-
Indole	+	+	Phenylalanine	-	-	Acetoin (VPT)	+/-	+/-

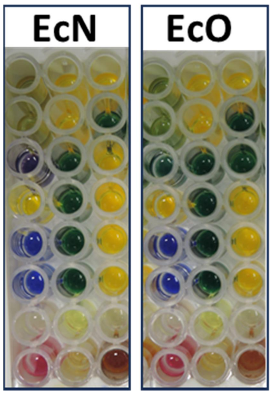

B

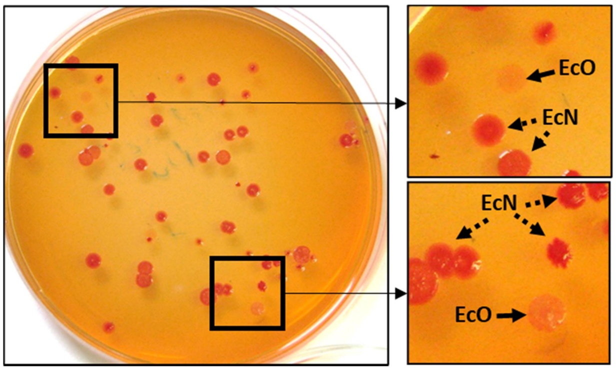

C

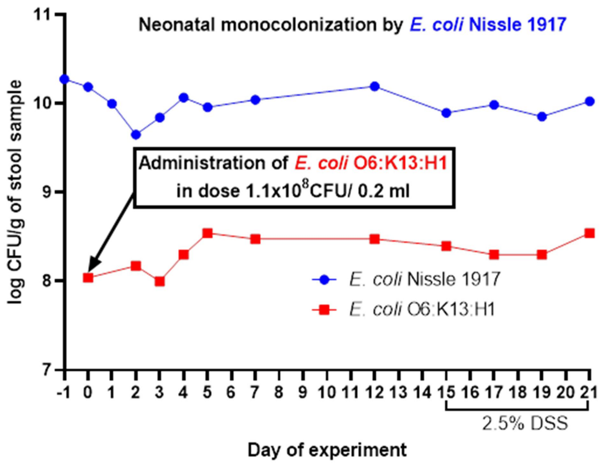

D

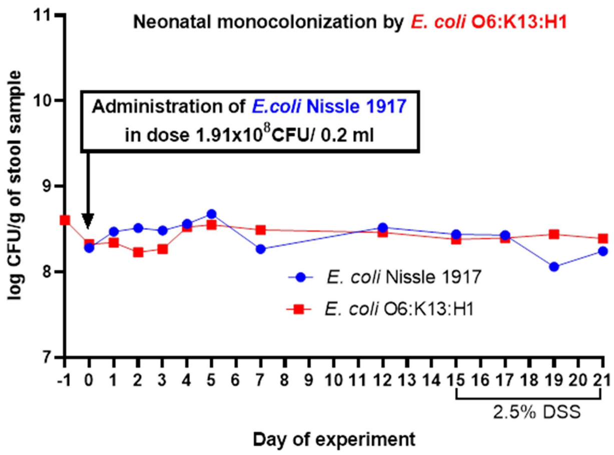

Figure S2: Strain specific detection of *E. coli* Nissle (EcN) and *E. coli* O6:K13:H1 (EcO) by cultivation method. **(A)** Determination of different strain-dependent raffinose utilization by ENTEROtest 24. **(B)** Representative picture of bacterial colony differentiation on modified McConkey agar supplemented by 2% raffinose (EcN red colonies; EcO pink colonies). **(C), (D)** Determination of EcN and EcO concentration in feces of experimental mice on modified McConkey agar plates supplemented by 2% raffinose.

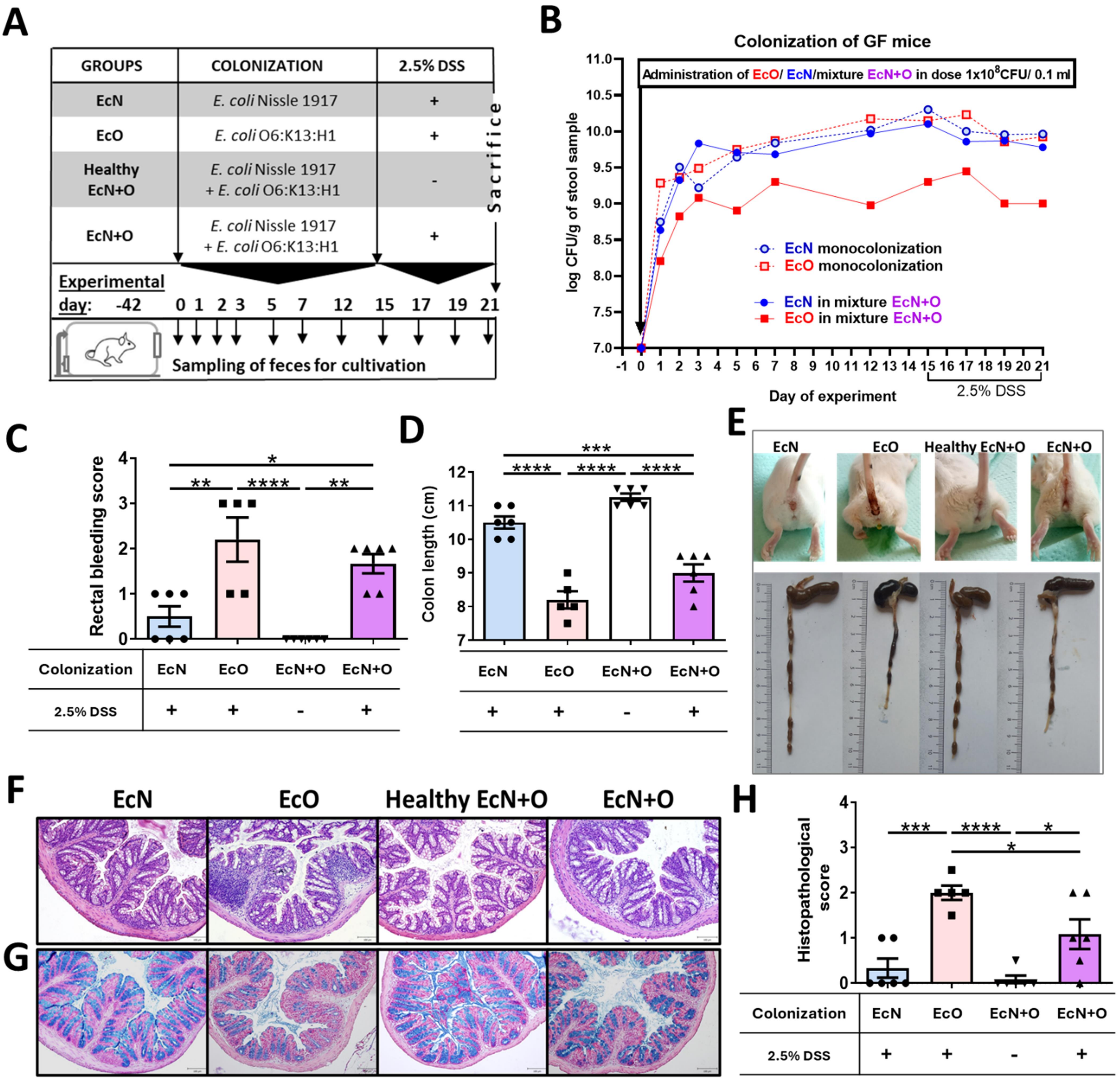

Figure S3: Colonization of adult germ-free mice by *E. coli* Nissle 1917, *E. coli* O6:K13:H1 and their mixture specifically modulate the development experimental colitis. (A) Experimental design. Six-week-old germ-free BALB/c mice were colonized with *E. coli* Nissle 1917 (EcN, n = 6), *E. coli* O6:K13:H1 (EcO, n = 5) or with their mixture (EcN+O, n = 6). Fifteen days after the co-colonization, intestinal inflammation was induced by 2.5% DSS administration in drinking water for 7 consecutive days. Mice colonized by EcN+O mixture without DSS treatment served as healthy control (healthy EcN+O, n = 6). **(B)** Fecal samples for detection of bacteria were collected throughout whole experiment at indicated days, serially diluted and plated on modified McConkey agar plates supplemented with 2% raffinose. The concentration of EcN or EcO bacterial load in feces of mono- (EcN or EcO alone) or bi-colonized (EcN+O mixture) mice is shown as log CFU/g of stool sample. **(C)** Rectal bleeding score was analyzed in mice colonized by EcN, EcO or their mixture after the treatment by 2.5% DSS solution to induce intestinal inflammation. **(D)** Shortening of the colon was evaluated in

experimental mice at sacrifice day. **(E)** Representative pictures of rectal bleeding and shortening of colon length in experimental mice. **(F)** Representative colon descendens cross-sections stained by hematoxylin and eosin (magnification 200x, scale 100µm). **(G)** Colon descendens tissue cross-sections stained by alcian blue and nuclear fast red are shown for visualization of mucus production (magnification 200x, scale 100µm). **(H)** Histopathological score was determined in colon tissue sections in 10%formaldehyde-fixed and paraffin embedded sections. One-way ANOVA with Tukey's post-test was used for determination of statistical differences (*p < 0.05, **p < 0.01, ***p < 0.001, ****p < 0.0001).
