# Supplementary material for: Priority order of neonatal colonization by a probiotic or pathogenic Escherichia coli strain dictates the host response to experimental colitis
Source: Front Microbiol. 2024 Aug 14;15:1393732. doi: 10.3389/fmicb.2024.1393732 (PMC11349737; doi:10.3389/fmicb.2024.1393732)
Supplement: Supplementary file 2 [file Data_Sheet_2.docx]

**SUPPLEMENTARY MATERIALS AND METHODS**

**Differentiation and cultivation of *E. coli* Nissle and *E. coli* O6:K13:H1 on modified McConkey agar**

Overnight cultures of *E. coli* Nissle and *E. coli* O6:K13:H1 were used for metabolic differentiation analysis by ENTEROtest 24 (Erba Lachema, Brno, Czechia) according to manufacturers´ instructions. Modified MacConkey agar was prepared as follows: 20.0 g/L of bacteriological peptone (Oxoid, Basingstoke, UK), 100 mL/L of 20% raffinose (Sigma-Aldrich, CO., St. Louis, USA), 1.5 g/L of bile salt No. 3 (Oxoid), 5.0 g/L of sodium chloride (Penta Chemicals, Brno, Czechia), 0.03 g/L of neutral red (Sigma-Aldrich), 0.5 g/L of crystal violet (Sigma-Aldrich), and 15.0 g/L of bacteriological agar No. 1 (Oxoid) were dissolved in distilled water, boiled, the pH 7.2 was adjusted and agar was sterilized by autoclaving (Splichalova et al., 2014). Petri dishes with modified McConkey agar were used for determination of CFU concentration of *E. coli* strains in serially diluted fecal samples of colonized mice at depicted days of experiment.

**EcN-, EcO-monocolonization and EcN+O-bi-colonization of adult germ-free mice**

**Bacteria preparation**

Cryostocks of EcN and EcO in concentration 1x10^8^ CFU/100 µL were prepared from overnight bacteria culture in LB broth (Oxoid) mixed with 30% glycerol and store at -80°C. The bacteria concentration was determined by serial dilution of cryostocks plating on LB agar. Before administration, the cryostocks were diluted to the working concentration of 1x10^8^ CFU/100 µL using sterile PBS.

**Mice**

Germ-free BALB/c female mice were kept under sterile conditions in Trexler-type plastic isolators in rooms with 12h light-dark cycle at 22°C, fed with 50 kGγ-irradiated sterile pellet diet (Altromin, Lage, Germany) and sterile drinking water *ad libitum*. Sterility was controlled every two-weeks as mentioned in Material and methods section. At the age of six weeks, the mice were divided into 4 groups and colonized by intragastric gavage of *E. coli* Nissle 1917 (EcN, n=6) or *E. coli* O6:K13:H1 (EcO, n=5) in concentration 1x10^8^ CFU/100 µL; or bi-colonized by mixture of EcN and EcO (EcN+O, n=6; healthy EcN+O, n=6), both strains in concentration 1x10^8^ CFU/100 µL. Then, mice were sterilely transferred to IsoCage system (Tecniplast S.P.A., Italy) with 12h light-dark cycle at 22°C, fed with 50 kG γ-irradiated sterile pellet diet (Altromin, Lage, Germany) and drinking water *ad libitum*. After 14 days, mice received autoclaved 2.5 % w/v dextran sulfate sodium (DSS, M.W. 36-50 kDa; MP Biomedicals, Illkirch, France) in drinking water *ad libitum* for seven consecutive days to induce acute colitis (see Supplementary figure S2A, experimental design). Six mice colonized by EcN+O without DSS-treatment served as healthy controls (healthy EcN+O group, n=6).

**Evaluation of intestinal inflammation**

At the end of experiment, occult bleeding was evaluated in stool samples by hemocult test (Hemocare, Care diagnostic GmbH, Austria). The colon was aseptically removed, the length was measured, and segments of colon tissue were fixed by 10% formaldehyde, transferred into 80% ethanol, embedded in paraffin, and stained as described in Material and method section. Data are expressed as mean ± standard error of the mean (SEM). Data were analyzed by one-way ANOVA with Tukey´s post-hoc test. Statistical analysis was performed using GraphPad Prism 8.0 Software (San Diego, CA, USA).

**References**

Splichalova, A., Splichal, I., Sonnenborn, U., & Rada, V. (2014). A modified MacConkey agar for selective enumeration of necrotoxigenic *E. coli* O55 and probiotic *E. coli* Nissle 1917. J Microbiol Methods. 104:82-86. doi:10.1016/j.mimet.2014.06.017.
